# Short-term exposure to fine particulate matter and genome-wide DNA methylation in chronic obstructive pulmonary disease: A panel study conducted in Beijing, China

**DOI:** 10.3389/fpubh.2022.1069685

**Published:** 2023-01-05

**Authors:** Ruirui Duan, Hongtao Niu, Fen Dong, Tao Yu, Xuexin Li, Hanna Wu, Yushi Zhang, Ting Yang

**Affiliations:** ^1^Department of Pulmonary and Critical Care Medicine, China-Japan Friendship Hospital, Beijing, China; ^2^Peking Union Medical College, Chinese Academy of Medical Sciences, Beijing, China; ^3^Peking University China-Japan Friendship School of Clinical Medicine, Beijing, China

**Keywords:** PM_2.5_, DNA-methylation, chronic obstructive pulmonary disease (COPD), health effect, inflammation

## Abstract

**Background:**

Fine particulate matter (PM_2.5_) is a crucial risk factor for chronic obstructive pulmonary disease (COPD). However, the mechanisms whereby PM_2.5_ contribute to COPD risk have not been fully elucidated. Accumulating evidence suggests that epigenetics, including DNA methylation, play an important role in this process; however, the association between PM_2.5_ exposure and genome-wide DNA methylation in patients with COPD has not been studied.

**Objective:**

To evaluate the association of personal exposure to PM_2.5_ and genome-wide DNA methylation changes in the peripheral blood of patients with COPD.

**Methods:**

A panel study was conducted in Beijing, China. We repeatedly measured and collected personal PM_2.5_ data for 72 h. Genome-wide DNA-methylation of peripheral blood was analyzed using the Illumina Infinium Human Methylation BeadChip (850 k). A linear-mixed effect model was used to identify the differentially methylated probe (DMP) associated with PM_2.5_. Finally, we performed a functional enrichment analysis of the DMPs that were significantly associated with PM_2.5_.

**Results:**

A total of 24 COPD patients were enrolled and 48 repeated DNA methylation measurements were associated in this study. When the false discovery rate was < 0.05, 19 DMPs were significantly associated with PM_2.5_ and were annotated to corresponding genes. Functional enrichment analysis of these genes showed that they were related to the response to toxic substances, regulation of tumor necrosis factor superfamily cytokine production, regulation of photosensitivity 3-kinase signaling, and other pathways.

**Conclusion:**

This study provided evidence for a significant relationship between personal PM_2.5_ exposure and DNA methylation in patients with COPD. Our research also revealed a new biological pathway explaining the adverse effects of PM_2.5_ exposure on COPD risk.

## 1. Introduction

Chronic obstructive pulmonary disease (COPD) is a heterogeneous disease characterized by persistent respiratory symptoms and airflow limitations. COPD is caused by the interaction between environmental and genetic risk factors (1). Fine particulate matter (PM_2.5_) is an important environmental risk factor for COPD. Epidemiological and clinical studies have shown that PM_2.5_ can exacerbate symptoms and accelerate lung function decline in patients with COPD and is an important risk factor for COPD and illness-associated mortality (2–6). Toxicological studies have shown that inflammation and oxidative stress are the key mechanisms whereby PM_2.5_ contributes to disease risk (7, 8). However, the potential biological pathways underscoring these relationships are far from clear.

Epigenetics, as a bridge linking environment-gene interactions, may provide a new biological mechanism for the adverse health effects of PM_2.5_ (9). Epigenetics describes changes in gene expression through DNA modification without changing the DNA sequence, including DNA methylation, histone modification, and non-coding RNA modification (10). Among them, DNA methylation is the most extensively studied and fully understood epigenetic modification. Abnormal DNA methylation patterns lead to the destruction of DNA integrity and gene expression changes (11).

External environmental factors, including air pollutants, can affect the level of DNA methylation, which in turn may alter gene expression profiles (12). Early studies provided evidence for the impact of air pollution on global DNA methylation by using long interspersed nucleotide element-1 (LINE-1) and Alu repetitive elements or the level of 5-methylcytosine (5 mC) in the genome (13–16). In addition, candidate-gene methylation analyses have yielded evidence suggesting that PM_2.5_ exposure is associated with CpG site methylation of genes such as *TNF-*α, *sICAM-1, sCD40L, interleukin 6*, and *inducible nitric oxide synthase gene* (*iNOS*) (9, 17–19). These results indicate that PM_2.5_ exposure reduces DNA methylation of specific gene loci, which are associated with the regulation of related gene expression.

Recently, several cohort studies used DNA methylation chip technology to explore the impact of air pollution exposure on genome-wide DNA methylation patterns and found that several CpG sites were related to air pollution exposure. The Women's Health Initiative and The Atherosclerosis Risk in Communities cohort studies found that DNA methylation at three CpG sites was significantly associated with PM exposure, and these sites were located in genes related with neurological, pulmonary, endocrine, and cardiovascular diseases (20). The UK LifeLines cohort study found that differential DNA methylation at seven CpG sites was significantly associated with NO_2_ exposure, of which two CpGs were considered potential mediators of the association between NO_2_ exposure and lung function (cg14938677 and cg18379295). NO_2_ exposure may thus affect lung function by changing DNA methylation patterns. (21) Panel studies conducted in Europe and China found that short-term exposure to PM_2.5_ was associated with changes in the methylation of multiple CpG sites in healthy non-smokers. Mediation analysis revealed that DNA methylation may play an important role in mediating the impact of PM_2.5_ on inflammatory pathways (17, 22, 23).

However, the relationship between PM_2.5_ exposure and peripheral blood genome-wide DNA methylation changes in patients with COPD remains unclear. Therefore, this study examined the association of PM_2.5_ and DNA methylation using the Illumina Infinium Human Methylation BeadChip (850 K) technology. We anticipated that this study might provide evidence for further exploration of the potential pathogenic mechanisms whereby PM_2.5_ contributes to COPD risk.

## 2. Materials and methods

### 2.1. Study design

This is a panel study conducted in Beijing, China, between July 2017 and August 2019, which is part of the research that has been reported (24). We collected repeated measurements of personal PM_2.5_ exposure and health outcomes in patients with COPD who were followed up every 3 months for a total of four visits in 1 year. Briefly, a personal PM_2.5_-monitoring device was worn for 3 days at each follow-up to obtain the PM_2.5_-exposure data, and peripheral blood samples were collected. Information about individual demographic and medical characteristics such as sex, age, body mass index (BMI), medication use, duration of COPD, and the number of acute exacerbations was collected at the baseline visit. Written informed consent was obtained from each participant before participation. The study protocol was approved by the Ethics Committee of the China-Japan Friendship Hospital (2017-19). This study was conducted following the principles of the Declaration of Helsinki.

### 2.2. Populations

Participants were recruited from the outpatient department of the China-Japan Friendship Hospital. The inclusion criteria were as follows: (1) age 45–75 years; (2) COPD definitively diagnosed by a professional doctor according to the Global Initiatives for Chronic Obstructive Pulmonary Disease guidelines (25); (3) patients who never smoked or quit smoking for more than 6 months.

The exclusion criteria, designed to reduce some factors that may have an obvious impact on DNA methylation, were as follows: (1) patients with a medical history of comorbidities such as malignant tumors, severe cardiovascular and cerebrovascular diseases, hepatic and renal insufficiency, active tuberculosis, and neuropsychiatric disorders; (2) pregnant or lactating women; and (3) alcoholics. Ultimately, 24 patients were enrolled in the study.

### 2.3. PM_2.5_ exposure assessment

The MicroPEM Personal Exposure Monitor (Version 3.2, RTI International, USA) was used to obtain personal PM_2.5_ exposure data during each follow up. In addition to monitoring the concentration of PM_2.5_ in real time, this instrument can detect the temperature and humidity of the surrounding environment. Its principle has been introduced in many other studies as well as in our prior research (24, 26). This instrument is small enough to fit in a backpack, and patients need to keep this backpack on their body during follow-up to continuously measure the PM_2.5_ exposure level for 72 h. Finally, we took the 72 h average as the personal PM_2.5_ exposure level during each follow-up period.

### 2.4. Blood sample collection and DNA extraction

After each PM_2.5_ exposure monitoring, 4 mL of fasting venous blood was collected from the patient, processed, and transferred to −80°C within 2 h. Genomic DNA was extracted from peripheral blood according to the manufacturer's protocol for methylation analysis. Briefly, DNA was isolated from peripheral blood using a DNeasy Blood and Tissue Kit (Qiagen, Hilden, Germany). The purity and concentration of the DNA were estimated using a Nanodrop 2000 (Thermo Fisher Scientific Inc., USA).

### 2.5. Genome-wide DNA methylation analysis

In this study, we performed DNA methylation analysis from two blood samples with the highest and lowest PM_2.5_ exposure in each patient during the four follow-up visits due to the budget constraints, which is a reference to previous panel study conducted by Mostafavi et al. (22) and a cross-over study conducted by Jiang et al. (27). The Illumina Infinium Human Methylation BeadChip (850 K) was used to analyze genome-wide DNA methylation. The 850 K microarrays can detect 866,895 cytosine methylation sites, covering CpG islands, enhancers, gene promoters, and coding regions in the human genome. Briefly, 500 ng of DNA from each sample was used for bisulfite conversion using an EZ DNA Methylation Kit (Zymo Research, USA). The converted products were then placed into 850 K BeadChips following the manufacturer's instructions and protocol. The data were mainly analyzed using the ChAMP package in R. For data quality control, we removed probes with a detection *p*-value >0.01, probes with less than three beads in at least 10% of samples, non-CpG probes, probes located on chromosomes X and Y, and (SNP-related probes) in turn. We used the β value to show the percentage of methylated cytosines at a given CpG site. The β-value is a continuous variable ranging from 0 to 1, representing the ratio of the intensity of the methylated probe signal to the total locus signal intensity. Then β-value matrix was normalized using BMIQ to adjust type I and type II probe bias (28). In addition, based on DNA methylation data, the Hausman algorithm was used to estimate the relative proportion of five different leukocyte types (i.e., CD8^+^T lymphocytes, CD4^+^T lymphocytes, natural killers cells, B cells, monocytes, and granulocytes) in each sample (29).

### 2.6. Enrichment analysis

To further evaluate the biological function of PM_2.5_-related unique CpG methylation sites, we conducted Gene Ontology (GO) enrichment using GenoGo Metacore. In addition, pathway analysis was performed based on the Kyoto Encyclopedia of Genes and Genomes (KEGG) database. *P*-values < 0.05 was considered significant.

### 2.7. Replication

To replicate our DMPs with results from previous clinical studies, we performed a systematic review in Pubmed (https://www.ncbi.nlm.nih.gov/pubmed/) from its inception to November 2022. We used the following search terms strategy:(((PM_2.5_) OR (Fine particulate matter)) OR (PM)) AND ((genome-wide DNA methylation) OR (epigenome-wide)). As no clinical study have been found on PM_2.5_ exposure and genome-wide DNA methylation in COPD patients currently. In order to verify the correlation between the DMPs and COPD disease, we looked up the DMPs in genome-wide DNA methylation studies of lung tissue in COPD patients (30). We set the cutoff of false discovery rate (FDR) < 0.05 for statistical significance for the look-up.

### 2.8. Statistical analysis

We used a linear mixed effects model to evaluate the impact of short-term exposure to PM_2.5_ on genome-wide DNA methylation. In this model, the random intercept for each patient to account for intra-individual correlation between repeated measurements, PM_2.5_ was regarded as the fixed-effect term. To control for potential confounding, we adjusted some fixed-effect covariates, such as sex, age, BMI, smoking status, temperature, relative humidity, and estimated leukocyte cell-type proportions. In addition, we controlled pulmonary function (FEV_1_%) in another model to exclude the influence of different severity of COPD disease itself. Finally, we performed the two-pollutant model to assess whether the estimated effects of PM_2.5_ are dependent on other air pollutants including carbon monoxide(CO), sulfur dioxide(SO_2_), ozone(O_3_) and nitrogen dioxide(NO_2_). Air pollutant data were obtained from environmental monitoring stations closest to the patients' home address. The average daily pollutants in the 3 days before blood sample collection were included in the two-pollutant model for analysis. The LME model was conducted using R software (version 4.0.0) with the package of “*lmertest*.” To control for type I errors in the genome-wide association, we used the Benjamini–Hochberg false discovery rate method (BH-FDR) to adjust the obtained *p*-values, and FDR < 0.05 was considered significant.

## 3. Results

### 3.1. Characteristics of study participants

A total of 18 males and 6 females with an average disease duration of 6.0 ± 4.7 years were enrolled in this study. Basic information on the participants included in this study is shown in Table 1. The mean age of the participants was 62.2 ± 8.0 years, and the mean BMI was 26.2 ± 6.7 kg/m^2^. Among them, 66.7% were former smokers, with an average smoking index of 25.6 packs/year. The average FEV_1_/FVC ratio and FEV_1_% after inhalation of the bronchodilator were 57.4 and 51.8%, respectively. Common comorbidities included hypertension, diabetes, and hyperlipidemia.

**Table 1:** Basic information of the participants.

**Characteristic**	
Male (*N*%)	18 (75%)
Age (years)	62.2 ± 8.0
BMI (kg/m^2^)	26.2 ± 6.7
FEV_1_/FVC	57.4 ± 8.7
FEV_1_%	51.8 ± 11.2
**Smoking**	
Never smoker (*N*%)	8 (33.3%)
Former smoker (*N*%)	16 (66.7%)
Pack-years	27.2 ± 6.1
Durations (years)	6.0 ± 4.7
**Comorbidity**	
Hypertension (*N*%)	3 (12.5%)
Diabetes mellitus (*N*%)	4 (16.6%)
Hyperlipidemia (*N*%)	3 (12.5%)

Data are presented as mean ± standard deviation or counts and percentages.

BMI, body mass index; FEV_1_/FVC, ratio of forced expiratory volume in one second to forced vital capacity.

### 3.2. Personal PM_2.5_ exposure and meteorological data

The personal PM_2.5_ exposure, and meteorological data are shown in Table 2. During the study period, the average 72 h mean concentrations of personal PM_2.5_ exposure varied from 8.5 to 150 μg/m^3^, with a mean of 62 μg/m^3^. We conducted a correlation analysis between PM_2.5_ and meteorological data, and found no significant correlation between PM_2.5_, temperature, or humidity (Supplementary Table 1).

**Table 2:** Descriptive statistics for PM_2.5_ and meteorological data.

	**Mean**	**SD**	**Min**	**P_25_**	**P_50_**	**P_75_**	**Max**
PM_2.5_ (μg/m^3^)	62.0	48.1	8.5	16.0	52.6	100.6	150.0
RH (%)	24.1	3.3	19.3	22.0	22.8	27.0	30.2
T (°C)	37.0	12.7	14.2	28.1	35.0	46.5	57.4

PM_2.5_, particulate matter with aerodynamic diameters ≤ 2.5 μm; RH, Relative Humidity; T, Temperature; SD, Standard deviation; Min, Minimum value; Max, Maximum value.

### 3.3. Correlation analysis between PM_2.5_ and genome-wide DNA methylation

**Figure 1:**
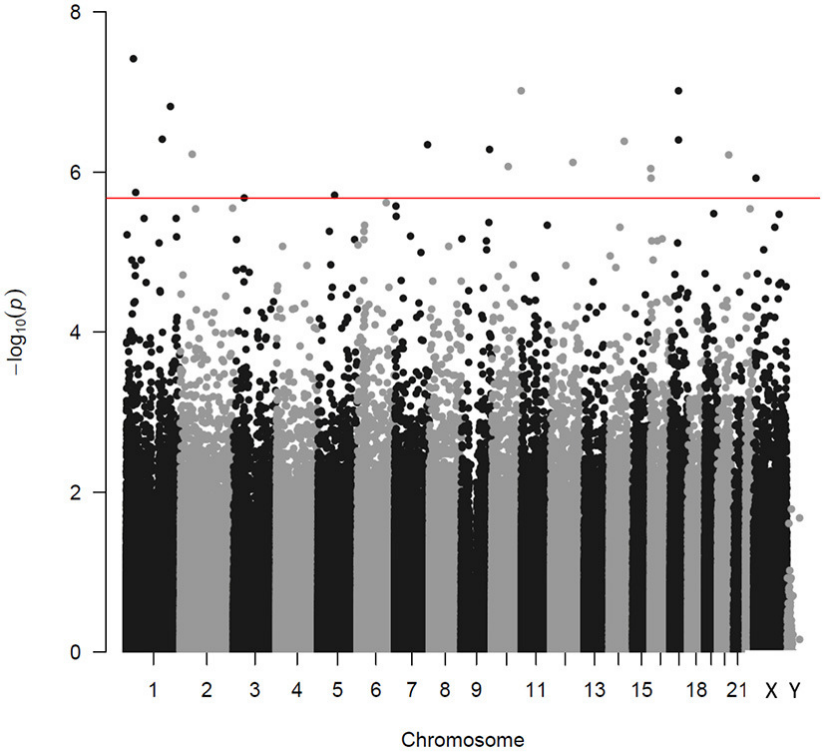
Manhattan plot indicating the associations between PM_2.5_ and DNA methylation. Every point corresponds to a CpG methylation site. The x-axis shows the chromosome of each CpG locus. The horizontal line corresponds to an adjusted *p*-value (FDR) of 0.05 level.

After removing probes that did not meet the quality control criteria, 840,157 CpGs were included in the LME model. Analyzing PM_2.5_ as a continuous variable, 19 DMPs were significantly associated with PM_2.5_ exposure under the condition of FDR < 0.05 (Figure 1). Among the 19 DMPs, 15 DMPs were negatively correlated with PM_2.5_. In other words, the methylation level decreased after exposure to PM_2.5_; four DMPs were positively correlated with PM_2.5_. The names of these DMPs, their corresponding gene names, their distribution, and location on gene structural elements, their chromosomes, and their correlation coefficients with PM_2.5_ are shown in Table 3. When FEV_1_% was adjusted in the LME model, 10 of the 19 DMP_S_ were still statistically significant, and the estimated effect coefficients did not change significantly (Table 4).

**Table 3:** Characteristics of DMPs associated with PM_2.5_ exposure in genome-wide methylation analysis, sorted by FDR.

**Probe ID**	**Coef^a^**	**SD**	***p*-value**	**FDR**	**Gene**	**Location**	**Chr**	**Relation to CpG_Island**
cg08772854	0.006	0.001	9.72E-08	0.002	INPP5A	Body	10	S_Shelf
cg23805785	−0.017	0.002	3.87E-08	0.002	IQCC	TSS200	1	Island
cg00017188	−0.016	0.002	9.73E-08	0.003	WIPF2	5'UTR	17	Island
cg18771855	−0.014	0.002	5.20E-07	0.006	C9orf16	TSS200	9	N_Shore
cg21212494	−0.013	0.001	1.51E-07	0.007	KLHL12	5'UTR	1	N_Shore
cg08455916	0.001	0.000	6.00E-07	0.007	C2orf73	Body	2	S_Shelf
cg00348771	−0.019	0.002	3.87E-07	0.009	MGST3	TSS1500	1	Island
cg12977244	0.005	0.001	6.09E-07	0.009	ZFP64	Body	20	S_Shelf
cg05469421	0.001	0.000	7.59E-07	0.010	ARL1	TSS1500	12	Island
cg27083176	−0.004	0.000	8.55E-07	0.023	USP54	Promoter	10	N_Shore
cg02376455	−0.010	0.001	3.95E-07	0.026	RARα	5'UTR	17	Island
cg07655457	−0.003	0.000	9.08E-07	0.028	HN1L	TSS200	16	Island
cg23781136	−0.017	0.002	4.10E-07	0.029	KCNK10	TSS200	14	Island
cg16251333	−0.014	0.002	1.19E-06	0.034	TCEANC	TSS200	X	Island
cg15447715	−0.008	0.001	4.51E-07	0.037	ABCB8	TSS200	7	Island
cg09879727	−0.012	0.001	1.20E-06	0.041	GFER	TSS200	16	Island
cg22613799	−0.007	0.001	1.81E-06	0.044	ZNF691	TSS200	1	N_Shore
cg03666316	−0.005	0.001	1.96E-06	0.046	F2R	Body	5	Island
cg03555424	−0.011	0.002	2.11E-06	0.048	APEH	TSS200	3	Island

^a^Regression coefficients, SD, and p-values were derived from the linear mixed-effect model. PM_2.5_, particulate matter with aerodynamic diameters ≤ 2.5 μm; FDR, false discovery rate; SD, standard deviation; DMP, differentially methylated probe; CpG, cytosine-guanine dinucleotide. Chr, Chromosome.

**Table 4:** Characteristics of DMPs associated with PM_2.5_ exposure in genome–wide methylation analysis after adjusted for lung function, sorted by FDR.

	**FEV**_**1**_ **% unadjusted**		**FEV**_**1**_ **% adjusted**	
**Probe ID**	**Coef** ±**SD**	**FDR**	**Coef** ±**SD**	**FDR**
cg08772854	0.006 ± 0.001^**^	0.002	0.006 ± 0.001^**^	0.001
cg23805785	−0.017 ± 0.002^**^	0.002	−0.016 ± 0.002^**^	0.001
cg00017188	−0.016 ± 0.002^**^	0.003	−0.016 ± 0.002^*^	0.024
cg18771855	−0.014 ± 0.002^**^	0.006	−0.014 ± 0.002^*^	0.019
cg08455916	0.001 ± 0.000^**^	0.007	0.001 ± 0.000^*^	0.019
cg21212494	−0.013 ± 0.001^**^	0.007	−0.012 ± 0.001^*^	0.03
cg00348771	−0.019 ± 0.002^**^	0.009	−0.019 ± 0.002^*^	0.022
cg12977244	0.005 ± 0.001^**^	0.009	0.005 ± 0.001^*^	0.035
cg05469421	0.001 ± 0.000^*^	0.01	0.001 ± 0.000^*^	0.024
cg27083176	−0.004 ± 0.000^*^	0.023	−0.004 ± 0.000	0.053
cg02376455	−0.01 ± 0.001^*^	0.026	−0.01 ± 0.001^*^	0.048
cg07655457	−0.003 ± 0.000^*^	0.028	−0.003 ± 0.000	0.073
cg23781136	−0.017 ± 0.002^*^	0.029	−0.017 ± 0.002	0.061
cg16251333	−0.014 ± 0.002^*^	0.034	−0.014 ± 0.002	0.11
cg15447715	−0.008 ± 0.001^*^	0.037	−0.008 ± 0.001	0.191
cg09879727	−0.012 ± 0.001^*^	0.041	−0.012 ± 0.001	0.102
cg22613799	−0.007 ± 0.001^*^	0.044	−0.007 ± 0.001	0.085
cg03666316	−0.005 ± 0.001^*^	0.046	−0.005 ± 0.001	0.113
cg03555424	−0.011 ± 0.002^*^	0.048	−0.011 ± 0.002	0.094

^*^FDR < 0.05, ^**^FDR < 0.01. FEV_1_%, forced expiratory volume in the 1st s; PM_2.5_, particulate matter with aerodynamic diameters ≤ 2.5 μm; FDR, false discovery rate; SD, standard deviation; DMP, differentially methylated probe ; CpG, cytosine–guanine dinucleotide.

Table 5 shows the estimated effect of other air pollutants on PM_2.5_-related DMPs in two-pollutant models. After adjusting for O_3_, only 3 of the 19 DMPs remained statistically significant, suggesting that O_3_ may neutralize or weaken the estimated effect of PM_2.5_ on DNA methylation. Similarly, 6 DMPs were still statistically significant after adjusting for CO. However, when NO_2_ and SO_2_ were adjusted in the LME model, most DMPs remained statistically significant (14 and 17 DMPs, respectively), and the estimated effect coefficients did not change significantly, suggesting that the estimated effect of PM_2.5_ on DNA methylation was relatively independent of NO_2_ or SO_2_.

**Table 5:** Two-pollutant models show the estimated effects of other air pollutants on PM_2.5_-related DMPs, sorted by FDR of PM_2.5_ alone.

	**PM**_**2.5**_		**PM**_**2.5**_ + **O**_**3**_		**PM**_**2.5**_ + **CO**		**PM**_**2.5**_ + **NO**_**2**_		**PM**_**2.5**_ + **SO**_**2**_	
**Probe ID**	**Coef** ±**SD**	**FDR**	**Coef** ±**SD**	**FDR**	**Coef** ±**SD**	**FDR**	**Coef** ±**SD**	**FDR**	**Coef** ±**SD**	**FDR**
cg08772854	0.006 ± 0.001^**^	0.002	0.007 ± 0.001^*^	0.016	0.008 ± 0.001^**^	0.003	0.007 ± 0.001^*^	0.032	0.007 ± 0.001^**^	0.000
cg23805785	−0.017 ± 0.002^**^	0.002	−0.017 ± 0.002^*^	0.026	−0.019 ± 0.002^**^	0.006	−0.018 ± 0.002^*^	0.025	−0.018 ± 0.002^**^	0.000
cg00017188	−0.016 ± 0.002^**^	0.003	−0.016 ± 0.002^*^	0.046	−0.019 ± 0.002^*^	0.011	−0.017 ± 0.002^*^	0.003	−0.017 ± 0.002^**^	0.001
cg18771855	−0.014 ± 0.002^**^	0.006	−0.016 ± 0.002	0.053	−0.017 ± 0.002^*^	0.018	−0.016 ± 0.002^*^	0.024	−0.015 ± 0.002^**^	0.002
cg08455916	0.001 ± 0.000^**^	0.007	0.001 ± 0.000	0.082	0.001 ± 0.000	0.166	0.001 ± 0.000	0.226	0.001 ± 0.000^*^	0.024
cg21212494	−0.013 ± 0.001^**^	0.007	−0.013 ± 0.002	0.142	−0.015 ± 0.002^*^	0.029	−0.013 ± 0.002^*^	0.028	−0.014 ± 0.001^**^	0.002
cg00348771	−0.019 ± 0.002^**^	0.009	−0.019 ± 0.003	0.087	−0.02 ± 0.003	0.092	−0.02 ± 0.003^*^	0.005	−0.02 ± 0.002^**^	0.002
cg12977244	0.005 ± 0.001^**^	0.009	0.004 ± 0.001	0.495	0.004 ± 0.001	0.529	0.005 ± 0.001	0.176	0.005 ± 0.001^*^	0.013
cg05469421	0.001 ± 0.000^*^	0.01	0.001 ± 0.000	0.108	0.002 ± 0.000	0.117	0.002 ± 0.000^*^	0.034	0.002 ± 0.000^**^	0.007
cg27083176	−0.004 ± 0.000^*^	0.023	−0.005 ± 0.001	0.239	−0.005 ± 0.001^*^	0.021	−0.005 ± 0.000^**^	0.008	−0.004 ± 0.001^*^	0.030
cg02376455	−0.01 ± 0.001^*^	0.026	−0.011 ± 0.001	0.334	−0.012 ± 0.002	0.108	−0.011 ± 0.001^*^	0.018	−0.011 ± 0.001^*^	0.016
cg07655457	−0.003 ± 0.000^*^	0.028	−0.004 ± 0.001	0.339	−0.004 ± 0.001	0.516	−0.003 ± 0.000	0.169	−0.003 ± 0.000	0.087
cg23781136	−0.017 ± 0.002^*^	0.029	−0.018 ± 0.003	0.417	−0.021 ± 0.003	0.057	−0.019 ± 0.002^*^	0.023	−0.018 ± 0.002^**^	0.005
cg16251333	−0.014 ± 0.002^*^	0.034	−0.014 ± 0.002	0.308	−0.016 ± 0.002	0.237	−0.014 ± 0.002^*^	0.027	−0.014 ± 0.002^*^	0.034
cg15447715	−0.008 ± 0.001^*^	0.037	−0.008 ± 0.001	0.472	−0.009 ± 0.001	0.579	−0.009 ± 0.001^*^	0.014	−0.009 ± 0.001^*^	0.031
cg09879727	−0.012 ± 0.001^*^	0.041	−0.013 ± 0.002	0.418	−0.014 ± 0.002	0.238	−0.013 ± 0.002	0.093	−0.013 ± 0.001^**^	0.009
cg22613799	−0.007 ± 0.001^*^	0.044	−0.007 ± 0.001	0.520	−0.007 ± 0.001	0.182	−0.007 ± 0.001	0.054	−0.007 ± 0.001^*^	0.026
cg03666316	−0.005 ± 0.001^*^	0.046	−0.005 ± 0.001	0.731	−0.006 ± 0.001	0.326	−0.005 ± 0.001	0.059	−0.005 ± 0.001	0.171
cg03555424	−0.011 ± 0.002^*^	0.048	−0.011 ± 0.002	0.711	−0.011 ± 0.002	0.135	−0.011 ± 0.002	0.460	−0.011 ± 0.002	0.072

^*^FDR < 0.05, ^**^ FDR < 0.01.

### 3.4. Enrichment analysis

**Figure 2:**
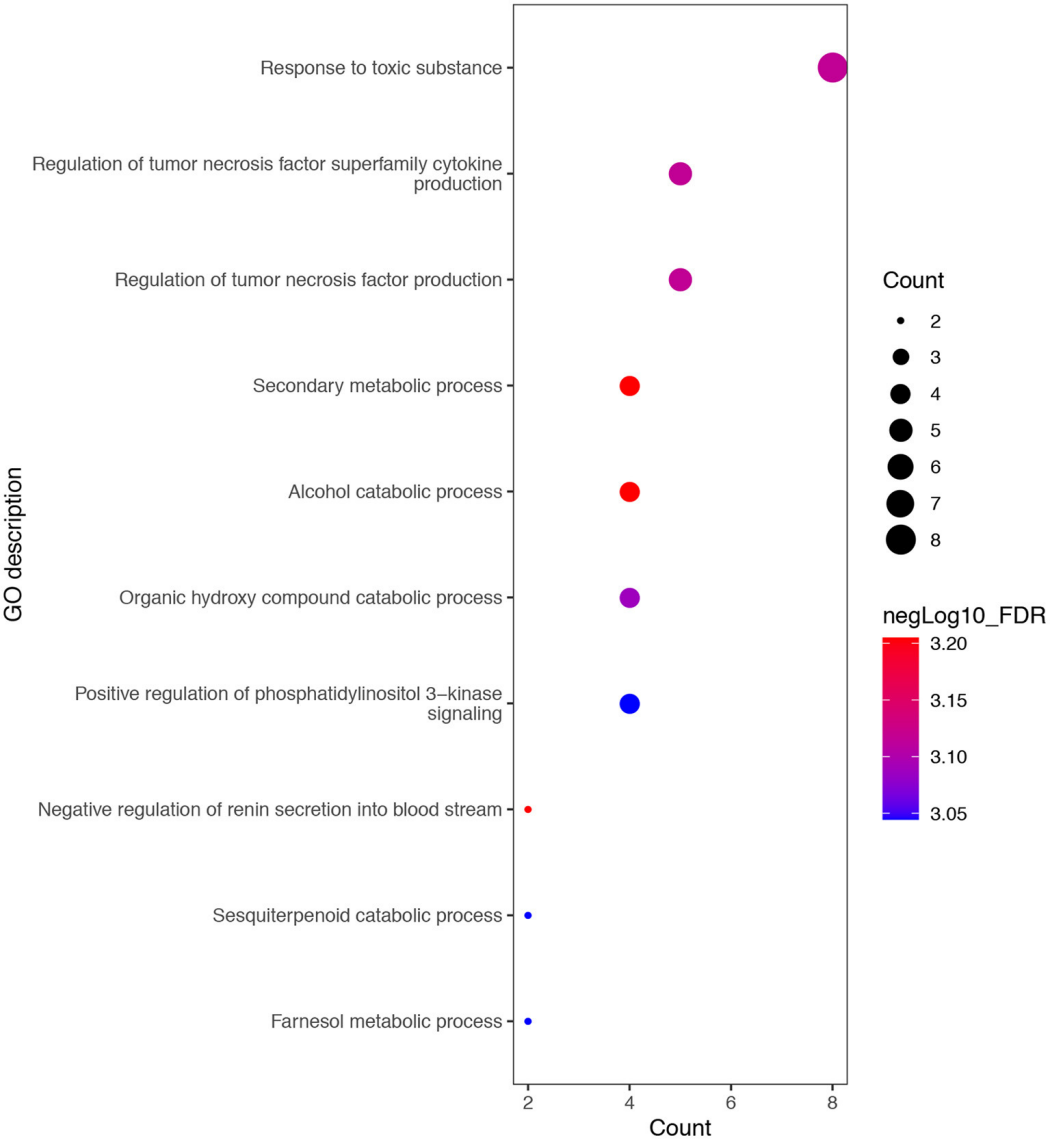
Top 10 GO pathways with the most significant differences. GO, Gene Ontology.

To evaluate the potential biological functions of the DMPs associated with PM_2.5_, we performed gene function enrichment analysis on these 19 DMPs. The ten most enriched GO pathways are shown in Figure 2, and the gene names involved in these GO pathways are shown in Supplementary Table 2. The GO enrichment mainly focused on cellular toxicity and metabolic responses to toxic substances, as well as the production of inflammatory factors, the response to toxic substances, regulation of tumor necrosis factor superfamily cytokine production, regulation of tumor necrosis factor production, secondary metabolic processes, organic hydroxy compound catabolic processes, and regulation of phosphatidylinositol 3-kinase signaling. In KEGG pathway enrichment analysis, a total of 30 pathways were involved in those DMPs, but only 3 of them reached statistical significance (*P*-values < 0.05). The three pathways are Pathogenic Escherichia coli infection, PI3K-Akt signaling pathway and Glutathione metabolism (Supplementary Table 3).

### 3.5. Replication of the previously published CpGs

We initially identified 51 manuscripts based on the search terms. After reading the full manuscripts, 15 genome-wide DNA methylation clinical studies were selected according to our analysis requirements (20–22, 27, 29, 31–40). Of the 19 DMPs associated with PM_2.5_ in our study, only one DMPs (cg08772854, mapped to INPP5A gene) was validate in the 2 of 15 previous genome-wide DNA methylation studies above (23, 32). And our results have the same direction effects as those 2 studies, the estimated effect coefficients are 0.006, 0.15, and 0.005, respectively (Supplementary Table 4). Moreover, cg08772854 was verified in the COPD cohort, indicating that cg08772854 is associated with COPD. The 18 remaining DMPs related to PM_2.5_ in our study were novel, meaning not reported to be associated with PM_2.5_ in other earlier studies.

## 4. Discussion

In this study, we used the latest genome-wide DNA methylation chip technology to explore the association between personal PM_2.5_ exposure and peripheral blood genome-wide DNA methylation in patients with COPD. We found that 19 DMPs were associated with PM_2.5_, and all 19 DMPs were annotated to specific genes. DNA methylation of these genes was reported for the first time to be related to exposure to PM_2.5_. Functional enrichment analysis showed that these genes were mainly involved in the metabolic response to external toxic substances and the production and regulation of inflammatory factors. To the best of our current knowledge, this is the first study to investigate the association of PM_2.5_ and epigenome-wide DNA methylation in COPD patients. The association of other air pollutants and DNA methylation in patients with COPD are also limited. Only a Korean cohort study (*N* = 100, which included 60 COPD patients) analyzed the effect of PM_10_ and NO_2_ on genome-wide DNA methylation (33). There was no duplication between our study and the latter. Therefore, this study provides new insights into the acute health effects of PM_2.5_ and COPD. We observed mostly negative estimated effect values between PM_2.5_ and genome-wide DNA methylation, in contrast to previous results that observed positive association between short-term PM exposure and DNA methylation levels at most DMPs (27, 32).

Inflammation and oxidative stress play important mediating roles in the effects of PM_2.5_ exposure on health. Some of the unique sites associated with PM_2.5_ exposure found in this study were genes associated with inflammation and oxidative stress. For example, we found that microsomal glutamate S-transfer 3 (mGST3) was hypo-methylation after PM_2.5_ exposure. mGST3 is a membrane-bound antioxidant enzyme with glutathione-dependent peroxidase activity, which is closely related to the antioxidant defense system and can protect mammals from oxidative stress damage caused by harmful substances in the environment (41). MGST3 plays an important role in protecting airway and alveolar epithelial cells by mitigating oxidative stress in the lung, which is also an important target in the treatment of COPD. Tang et al. (42) found that MGST3 was associated with FEV_1_/FVC in patients with COPD. In addition, mGST3 is involved in the catalysis of leukotriene C4 and reduced glutathione, thereby playing an important role in inflammation and oxidative stress. These findings suggest that PM_2.5_ can affect inflammation and oxidative stress in the lung by affecting the methylation status of genes related to inflammation and oxidative stress, thereby affecting the occurrence and development of COPD (43, 44).

Changes in gene methylation related to immune functions were also observed in this study. For example, Kelch-like family member 12 (KLHL12) and ZEP64 showed low methylation levels after exposure to PM_2.5_. KLHL12 is a nuclear protein that can specifically bind to the dopamine D4 receptor and participate in ubiquitination (45). KLHL12 is associated with a variety of autoimmune diseases. For example, KLHL12 has been identified as an autoantigen in Sjogren's syndrome and is a potential biomarker for primary biliary cirrhosis (46–48). Activation of Toll-like receptors (TLRs) is very important for initiating protective immune responses. Wang et al. found that overexpression of ZFP64 could significantly upregulate TLR-induced NF-κB activation and promote TNF-α, IL-6, and IFN-β production, while knockdown of ZFP64 expression significantly inhibited the production of these cytokines, indicating that ZFP64 can promote the activation of the TLR signaling pathway (49). The roles of these genes in COPD have not been studied, and further biological studies should be carried out to further explore the potential mechanisms of these genes in the pathogenesis of COPD.

We performed functional enrichment analysis of the genes where these 19 DMPs were located and provided biological implications for our findings. Significantly enriched pathways included responses to toxic substances, secondary metabolic processes, and other metabolic pathways to external toxic substances. PM_2.5_ not only contains harmful compounds such as polycyclic aromatic hydrocarbons, but also adsorbs other toxic substances on its surface. These toxic stimuli can cause changes in the state of cells, including gene expression. Our study showed that PM_2.5_ may stimulate the body's toxic and metabolic response by affecting the changes in DNA methylation patterns of genes involved in these pathways.

Functional enrichment analysis revealed that these 19 DMPs were also involved in inflammatory and oxidative stress-related pathways, e.g., the regulation of tumor necrosis factor production and phosphatidylinositol 3-kinase signaling (PI3K). Previous studies have shown that most members of the tumor necrosis factor superfamily can participate in inflammatory responses, regulate cell proliferation, differentiation, and apoptosis, and also play a role in various illnesses including cancer, cardiovascular disease, pulmonary conditions, and autoimmune disorders (50, 51). Moreover, members of the TNF superfamily can activate NF-κB, leading to a persistent inflammatory response in the lung. Multiple members of the tumor necrosis factor superfamily, such as tumor necrosis factor-related apoptosis-inducing ligand (TRAIL) (52), CD40L (53), and TNF-α (54), play an important role in the pathogenesis of COPD. The PI3K signaling pathway is an important signaling cascade involved in a variety of important pathophysiological processes, including oxidative stress and inflammatory responses. Several experimental studies have shown that PI3K signaling is significantly activated in COPD and plays a key role in chronic inflammation and oxidative stress in COPD (55, 56). In this study, functional enrichment analysis revealed the potential biological functions of aberrant DNA methylation genes related to PM_2.5._

Our study had several strengths. First, we obtained personal PM_2.5_ exposure data using an individual sampler, allowing us to more accurately assess exposure to PM_2.5_. Additionally, we used a panel study approach, allowing everyone to act as their own control factor, avoiding confusion over time-invariant factors. Simultaneously, our study had some limitations. First, the expensive and cumbersome assessment of individual PM_2.5_ exposure limited the sample size. Second, only differentially methylated genes were analyzed, and the expression levels of related genes were not measured or verified. In the future, mRNA and protein expression of these genes will be analyzed to provide evidence for the role of PM_2.5_ exposure-related DNA methylation in the development of COPD.

In conclusion, our study provides strong evidence suggesting that short-term exposure to PM_2.5_, which is involved in response to toxic substances, tumor necrosis superfamily cytokine production, oxidative stress, and other biological pathways, may lead to changes in the methylation status of multiple CpG sites in the peripheral blood of patients with COPD. Further studies with larger sample sizes are necessary to confirm the findings of this study.

## Ethics statement

The studies involving human participants were reviewed and approved by China-Japan friendship hospital. The patients/participants provided their written informed consent to participate in this study.

## Author contributions

TiY proposed this study and revised the manuscript. RD and HN completed participants' recruitment and follow-up. RD did the data analysis and completed the manuscript. TaY and XL collected samples and did quality control. All authors revised the report and approved the final version before submission.
