# Supplementary material for: Short-term exposure to fine particulate matter and genome-wide DNA methylation in chronic obstructive pulmonary disease: A panel study conducted in Beijing, China
Source: Front Public Health. 2023 Jan 5;10:1069685. doi: 10.3389/fpubh.2022.1069685 (PMC9850166; doi:10.3389/fpubh.2022.1069685)
Supplement: Supplementary file 1 [file Table_1.DOCX]

**Supplementary Table 1: Correlation coefficients between PM_2.5_ and meteorological data.**

|  | **PM_2.5_(μg/m^3^）** | **T (℃)** | **RH (%)** |
| --- | --- | --- | --- |
| **PM_2.5_(μg/m^3^)** | 1 | -0.24 | -0.23 |
| **T (℃)** |  | 1 | -0.16 |
| **RH (%)** |  |  | 1 |

Notes: Data are presented as correlation coefficients. T, temperature; RH, relative humidity.

**Supplementary Table 2: Genes involved in the GO pathway analyzed by enrichment of differentially methylated sites**

| **GO Name** | **P-value** | **Gene Name** |
| --- | --- | --- |
| [response to toxic substance](http://portal.genego.com/cgi/process.cgi?id=-824870215) | 7.654E-04 | GPCRs, MGST3, ALR1, AK1BA, GFER, RARα |
| [regulation of tumor necrosis factor production](http://portal.genego.com/cgi/process.cgi?id=-1498632903) | 7.654E-04 | GPCRs, MGST3, RAR, ZFP64, RARα |
| [regulation of tumor necrosis factor superfamily cytokine production](http://portal.genego.com/cgi/process.cgi?id=-575686204) | 7.654E-04 | GPCRs, MGST3, RAR, ZFP64, RARα |
| [secondary metabolic process](http://portal.genego.com/cgi/process.cgi?id=-993055466) | 6.238E-04 | ARL1, MGST3, AK1BA |
| [alcohol catabolic process](http://portal.genego.com/cgi/process.cgi?id=-1721729403) | 6.238E-04 | GPCRs, ALR1, AK1BA, INPP5A |
| [organic hydroxy compound catabolic process](http://portal.genego.com/cgi/process.cgi?id=-600030743) | 8.175E-04 | GPCRs, ALR1 AK1BA, INPP5A |
| [positive regulation of phosphatidylinositol 3-kinase signaling](http://portal.genego.com/cgi/process.cgi?id=-784380377) | 9.028E-04 | F2R, GPCRs, RARα |
| [negative regulation of renin secretion into blood stream](http://portal.genego.com/cgi/process.cgi?id=-1209486082) | 6.238E-04 | F2R, GPCRs |
| [sesquiterpenoid catabolic process](http://portal.genego.com/cgi/process.cgi?id=-597858772) | 9.028E-04 | ALR1, AK1BA |
| [farnesol metabolic process](http://portal.genego.com/cgi/process.cgi?id=-129294879) | 9.028E-04 | ALR1, AK1BA |

| **KEGG Pathway Name** | **P-value** | **Gene Name** |
| --- | --- | --- |
| Pathogenic Escherichia coli infection | 0.011 | WIPF2/F2R |
| PI3K-Akt signaling pathway | 0.038 | F2R |
| Glutathione metabolism | 0.048 | MGST3 |
| Acute myeloid leukemia | 0.057 | RARA |
| Drug metabolism - cytochrome P450 | 0.061 | MGST3 |
| Inositol phosphate metabolism | 0.062 | INPP5A |
| Platinum drug resistance | 0.062 | MGST3 |
| Gastric acid secretion | 0.064 | KCNK10 |
| Metabolism of xenobiotics by cytochrome P450 | 0.066 | MGST3 |
| Drug metabolism - other enzymes | 0.067 | MGST3 |
| Chemical carcinogenesis | 0.070 | MGST3 |
| Complement and coagulation cascades | 0.071 | F2R |
| Phosphatidylinositol signaling system | 0.081 | INPP5A |
| Th17 cell differentiation | 0.089 | RARA |
| Platelet activation | 0.103 | F2R |
| Insulin signaling pathway | 0.113 | INPP5A |
| Yersinia infection | 0.113 | WIPF2 |
| Estrogen signaling pathway | 0.114 | RARA |
| Fluid shear stress and atherosclerosis | 0.114 | MGST3 |
| Phospholipase D signaling pathway | 0.121 | F2R |
| Hepatocellular carcinoma | 0.137 | MGST3 |
| Transcriptional misregulation in cancer | 0.155 | RARA |
| Calcium signaling pathway | 0.162 | F2R |
| Rap1 signaling pathway | 0.168 | F2R |
| cAMP signaling pathway | 0.173 | F2R |
| Regulation of actin cytoskeleton | 0.174 | F2R |
| Endocytosis | 0.199 | WIPF2 |
| Neuroactive ligand-receptor interaction | 0.261 | F2R |
| ABC transporters | 0.269 | ABCB8 |

**Supplementary Table 3:** **Differential CpG methylation sites involved in the KEGG pathway and the related genes.**

**Supplementary Table 4: Replication of the previously published CpGs.**

| **ProbeID** | **Gene** | **Subject of Research** | **FDR** | **Coef** |
| --- | --- | --- | --- | --- |
| cg08772854* | INPP5A | PM_2.5_ and COPD | 0.002 | 0.006 |
| cg08772854^[1]^ | INPP5A | PM_2.5_ and adult | 0.00675 | 0.15 |
| cg08772854^[2]^ | INPP5A | PM_2.5_ and adult | 0.017 | 0.005 |
| cg08772854^[3]^ | INPP5A | COPD and non-COPD | 2.40E-08 | -0.033 |

Note: *Results in this study
